# Comparison of ^90^Y SIRT predicted and delivered absorbed doses using a PSF conversion method

**DOI:** 10.1016/j.ejmp.2021.07.026

**Published:** 2021-09

**Authors:** Allison J. Craig, Iain Murray, Ana M. Denis-Bacelar, Bruno Rojas, Jonathan I. Gear, Lucy Hossen, Annelies Maenhout, Nasir Khan, Glenn D. Flux

**Affiliations:** a Joint Department of Physics, Royal Marsden NHSFT, Sutton, United Kingdom; b The Institute of Cancer Research, London, United Kingdom; c National Physical Laboratory, Teddington, United Kingdom; d Royal Brompton & Harefield NHSFT, London, United Kingdom; e Chelsea & Westminster NHSFT, London, United Kingdom

**Keywords:** Radioembolisation, Y-90, SIRT, Liver, Dosimetry, Microspheres

## Abstract

**Purpose:**

The aims of this study were to develop and apply a method to correct for the differences in partial volume effects of pre-therapy Technetium-99 m (^99m^Tc)-MAA SPECT and post-therapy Yttrium-90 (^90^Y) bremsstrahlung SPECT imaging in selective internal radiation therapy, and to use this method to improve quantitative comparison of predicted and delivered ^90^Y absorbed doses.

**Methods:**

The spatial resolution of ^99m^Tc SPECT data was converted to that of ^90^Y SPECT data using a function calculated from ^99m^Tc and ^90^Y point spread functions. This resolution conversion method (RCM) was first applied to ^99m^Tc and ^90^Y SPECT phantom data to validate the method, and then to clinical data to assess the power of ^99m^Tc SPECT imaging to predict the therapeutic absorbed dose.

**Results:**

The maximum difference between absorbed doses to phantom spheres was 178%. This was reduced to 27% after the RCM was applied.

The clinical data demonstrated differences within 38% for mean absorbed doses delivered to the normal liver, which were reduced to 20% after application of the RCM. Analysis of clinical data showed that therapeutic absorbed doses delivered to tumours greater than 100 cm^3^ were predicted to within 52%, although there were differences of up to 210% for smaller tumours, even after the RCM was applied.

**Conclusions:**

The RCM was successfully verified using phantom data. Analysis of the clinical data established that the ^99m^Tc pre-therapy imaging was predictive of the ^90^Y absorbed dose to the normal liver to within 20%, but had poor predictability for tumours smaller than 100 cm^3^.

## Highlights

• The resolution conversion method allowed comparison of ^99m^Tc and ^90^Y imaging.
• ^99m^Tc pre-therapy imaging was predictive of the ^90^Y absorbed dose to normal liver.
• ^99m^Tc pre-therapy imaging had poor predictability for tumours smaller than 100 cm^3^.

## Introduction

Selective internal radiation therapy (SIRT) is used to treat Hepatocellular Carcinoma and liver metastases originating from other primary cancers. Yttrium-90 (^90^Y) microspheres are administered via a catheter directly into the hepatic artery where they are deposited within the hepatic arterioles surrounding the tumours. Pre-therapy imaging with Technetium-99 m (^99m^Tc) labelled macroaggregated albumin (MAA) is performed to assess any extra-hepatic uptake. This includes calculation of the lung shunt, a measure of the microsphere deposition within the lungs. Post-therapy imaging may be performed by ^90^Y PET or bremsstrahlung SPECT. ^90^Y PET has been shown to be more quantitatively accurate than bremsstrahlung SPECT [1], [2]. However, SPECT is more widely available and cost-effective [3]. Quantitative ^90^Y bremsstrahlung SPECT imaging is challenging due to the lack of a photopeak. It also has poor spatial resolution compared to ^99m^Tc SPECT imaging, with large tails in the ^90^Y point spread function (PSF) due to high energy bremsstrahlung photons penetrating the collimator septa [4]. Despite this ^90^Y bremsstrahlung imaging has been used for over 35 years for both planar and SPECT quantification [5], [6], [7], [8], [9], [10], [11].

^90^Y activity calculations and treatment planning may be based on the pre-therapy ^99m^Tc-MAA SPECT imaging, and rely on the assumption that the MAA particle distribution is identical to that of the ^90^Y-microsphere distribution. However, there are a number of reasons why this assumption may be flawed which include differences in the size and number of particles administered [12], the catheter position [13], [14], [15], administration flow rates [13], [14], [15] and regional blood flow differences between administrations [16]. Previous studies have compared the pre- and post-therapy imaging [14], [17], [18], [19], [20], [21], with the majority of these studies taking no account of the differences in the partial volume effect of the images [14], [17], [18], [19]. However, Gnesin et al [20] applied recovery coefficients to predicted and delivered absorbed doses to tumours derived from ^99m^Tc-MAA SPECT and ^90^Y PET images respectively, while a recent study by Mikell et al [21] convolved ^90^Y PET images with a Gaussian function to match the spatial resolution of ^99m^Tc-MAA SPECT images.

The aim of this study was to develop a resolution conversion method (RCM) to correct for the differences in partial volume effects between ^99m^Tc-MAA SPECT and ^90^Y bremsstrahlung SPECT images. The RCM converts the spatial resolution of the ^99m^Tc SPECT data to that of a post-therapy ^90^Y SPECT image by convolution with a function calculated from ^99m^Tc and ^90^Y point spread functions. This method was then applied retrospectively to patient data to assess the ability of quantitative ^99m^Tc-MAA SPECT to predict ^90^Y normal liver and tumour absorbed doses.

## Methods

### Resolution conversion method

A method, named the resolution conversion method (RCM), was developed to convert a reconstructed ^99m^Tc-MAA SPECT image, $I_{Tc99m}$, to the same spatial resolution as a reconstructed ^90^Y bremsstrahlung SPECT image. The RCM operates by the convolution of the ^99m^Tc reconstructed SPECT data with a function ($p_{Tc99m\to Y90}$) calculated from the measured ^99m^Tc and ^90^Y PSF’s,$p_{Tc99m}$ and $p_{Y90}$, respectively. $p_{Tc99m\to Y90}$ is defined such that:

(1) $$p_{Y90}=p_{Tc99m}⊗p_{Tc99m\to Y90}$$

$p_{Tc99m}$ is described as a Gaussian function with a standard deviation $\sigma_{Tc99m}$:

(2) $$p_{Tc99m}=\frac{1}{{\left(2\pi \right)}^{3/2}\;\sigma_{Tc99m}^{3}\;}e^{-\left[r^{2}/2\;\sigma_{Tc99m}^{2}\right]}$$

where

(3) $$r^{2}=x^{2}+y^{2}+z^{2}$$

and

(4) $$\underset{-\infty }{\int^{\infty }}\underset{-\infty }{\int^{\infty }}\underset{-\infty }{\int^{\infty }}p_{Tc99m}{\mathrm{dr}}^{3}=1$$

where

(5) $${\mathrm{dr}}^{3}=dxdydz$$

The measured FWHM values were all shown to be within one voxel, thus $\sigma_{Tc99m}$ is assumed to be the same in the x, y and z directions.

$p_{Y90}$ cannot be adequately described by using a single Gaussian function due to the presence of large septal penetration tails [4]. Therefore $p_{Y90}$ is described by the sum of two Gaussian functions (${p_{Y90}}_{1}$ and ${p_{Y90}}_{2}$):

(6) $$p_{Y90}={p_{Y90}}_{1}+{p_{Y90}}_{2}$$

where ${p_{Y90}}_{1}$ and ${p_{Y90}}_{2}$ are two independent functions given by:

(7) $$p_{Y90_{1}}=\frac{A_{1}}{{\left(2\pi \right)}^{3/2}\;\sigma_{Y90_{1}}^{2}}e^{-\left[r^{2}/2\sigma_{Y90_{1}}^{2}\right]}$$

(8) $$\;p_{Y90_{2}}=\frac{A_{2}}{{\left(2\pi \right)}^{3/2}\;\sigma_{Y90_{2}}^{2}}e^{-\left[r^{2}/2\sigma_{Y90_{2}}^{2}\right]}$$

$\sigma_{{Y90}_{1}}$and $\sigma_{{Y90}_{2}}$ are the standard deviations describing the respective Gaussian distribution functions, and $A_{1}$ and $A_{2}$ are constants derived from the integrals of the Gaussian distributions under the constraint that:

(9) $$\underset{-\infty }{\int^{\infty }}\underset{-\infty }{\int^{\infty }}\underset{-\infty }{\int^{\infty }}p_{Y90}{\mathrm{dr}}^{3}=1$$

As $p_{Y90}$ consists of two independent functions, the same must be true for $p_{Tc99m\to Y90}$, and each of these can be convolved separately with $p_{Tc99m}$. Therefore, $p_{Tc99m\to Y90}$ may be expressed as:

(10) $$p_{Tc99m\to Y90}={p_{Tc99m\to Y90}}_{1}+{p_{Tc99m\to Y90}}_{2}$$

where

(11) $$p_{Tc99m\to Y90_{1}}=\frac{B_{1}}{{\left(2\pi \right)}^{3/2}\;\sigma_{Tc99m\to Y90_{1}}^{3}\;}e^{-\left[r^{2}/2\sigma_{Tc99m\to Y90_{1}}^{2}\right]}$$

and

(12) $$p_{Tc99m\to Y90_{2}}=\frac{B_{2}}{{\left(2\pi \right)}^{3/2}\;\;\sigma_{Tc99m\to Y90_{2}}^{3}}e^{-\left[r^{2}/2\sigma_{Tc99m\to Y90_{2}}^{2}\right]}$$

with standard deviations $\sigma_{{Tc99m\to Y90}_{1}}$ and $\sigma_{{Tc99m\to Y90}_{2}}$ and constants $B_{1}$ and $B_{2}$ such that:

(13) $$\underset{-\infty }{\int^{\infty }}\underset{-\infty }{\int^{\infty }}\underset{-\infty }{\int^{\infty }}{p_{Tc99m\to Y90}}_{1}{\mathrm{dr}}^{3}=\underset{-\infty }{\int^{\infty }}\underset{-\infty }{\int^{\infty }}\underset{-\infty }{\int^{\infty }}p_{{Y90}_{1}}{\mathrm{dr}}^{3}$$

(14) $$\underset{-\infty }{\int^{\infty }}\underset{-\infty }{\int^{\infty }}\underset{-\infty }{\int^{\infty }}{p_{Tc99m\to Y90}}_{2}{\mathrm{dr}}^{3}=\underset{-\infty }{\int^{\infty }}\underset{-\infty }{\int^{\infty }}\underset{-\infty }{\int^{\infty }}p_{{Y90}_{2}}{\mathrm{dr}}^{3}$$

The convolution of a Gaussian function with another Gaussian function results in a third Gaussian function with a variance equal to the sum of the variances in the original two functions [22]. The variances $\sigma_{{Y90}_{1}}^{2}$and $\sigma_{{Y90}_{2}}^{2}$ are then given by:

(15) $$\sigma_{{Y90}_{1}}^{2}=\sigma_{{Tc99m\to Y90}_{1}}^{2}+\sigma_{Tc99m}^{2}$$

(16) $$\sigma_{{Y90}_{2}}^{2}=\sigma_{{Tc99m\to Y90}_{2}}^{2}+\sigma_{Tc99m}^{2}$$

Thus the variances of $p_{Tc99m\to Y90}$ are defined as:

(17) $$\sigma_{{Tc99m\to Y90}_{1}}^{2}=\sigma_{{Y90}_{1}}^{2}-\sigma_{Tc99m}^{2}$$

(18) $$\sigma_{{Tc99m\to Y90}_{2}}^{2}=\sigma_{{Y90}_{2}}^{2}-\sigma_{Tc99m}^{2}$$

Thus the conversion of the ^99m^Tc SPECT image, $I_{Tc99m}$, to a representation of an image with a ^90^Y resolution, $I_{Tc99m\to Y90},$ is given by:

(19) $$I_{Tc99m\to Y90}=I_{Tc99m}\frac{B_{1}}{{\left(2\pi \right)}^{3/2}\;\;\sigma_{Tc99m\to Y90_{1}}^{3}\;}e^{-\left[r^{2}/2\sigma_{Tc99m\to Y90_{1}}^{2}\right]}+I_{Tc99m}\frac{B_{2}}{{\left(2\pi \right)}^{3/2}\;\;\sigma_{Tc99m\to Y90_{2}}^{3}\;}e^{-\left[r^{2}/2\sigma_{Tc99m\to Y90_{2}}^{2}\right]}$$

## Measurement of the point spread functions

A point source of ^99m^Tc was placed into the NEMA IEC phantom and a SPECT-CT acquisition was performed on a Siemens Symbia SPECT-CT system (Siemens Healthcare Limited, Erlangen, Germany), with the source centred in the x-y plane. The acquisition was then repeated with a point source of ^90^Y. SPECT acquisition parameters are detailed in Table 1. Non-circular orbits were performed for all scans to match with the standard clinical protocol.

**Table 1:** SPECT acquisition parameters.

	**^99m^Tc**	**^90^Y**
**Energy window (kev)**	129.5 – 150.5	71.25 – 118.75
**Collimator**	LEHR	MEGP
**No. of views per head**	64	64
**Time per projection (seconds)**	15 s for all SPECT scans	60 s for point source scan, 19 s for phantom and clinical scans
**Matrix size**	256 × 256	256 × 256
**Orbit**	Non-circular, step and shoot	Non-circular, step and shoot

### Reconstruction parameters

^99m^Tc SPECT acquisition data were reconstructed using an OSEM algorithm for 5 iterations, 8 subsets, CT attenuation correction and scatter correction (Hermes Medical Solutions, Hybrid Viewer). ^90^Y SPECT acquisition data were reconstructed for 5 iterations, 8 subsets and CT attenuation correction was performed for 95 keV, the mid-point of the ^90^Y energy window.

### Analysis of point spread function measurements

Profiles were drawn through the reconstructed image of the ^99m^Tc point source in three orthogonal planes (x, y, z) to obtain the PSF. Full width half maximum (FWHM) values were calculated for each plane using the method described by NEMA [23]. An average FWHM was used to calculate $\sigma_{Tc99m}$ for input into equations 17 and 18.

Voxel count values and positions were extracted from the reconstructed ^90^Y SPECT image from a cubic volume of interest (23 cm^3^) centred on the point source. A mathematical model, which consisted of the sum of two Gaussian functions (equations 6–8), was fitted to these data, using a method of least squares (Solver, Microsoft Excel (2010)) which iteratively minimises the sum of the square of the residuals between the fitted and measured data. Values of the fit parameters $\sigma_{{Y90}_{1}}$and $\sigma_{{Y90}_{2}}$were input into equations 17 and 18.

## Phantom study

### Phantom SPECT scans

A phantom study was undertaken to validate the RCM. The NEMA IEC phantom, with six spherical inserts of diameter: 37, 28, 22, 17, 13 and 10 mm, was filled with radioactivity to represent the normal liver background, whilst the spheres were filled with higher activities to represent tumours. Four clinically realistic sphere-to-background ratios were used (8:1, 6:1, 4:1 and 2:1) and a SPECT-CT acquisition was performed for each (Table 1). Acquisitions were repeated with the phantom and spheres containing solutions of ^90^Y. Total activities in the phantom ranged from 50 to 168 MBq for ^99m^Tc and 654–2465 MBq for ^90^Y. Acquisition data were reconstructed using the same reconstruction parameters used to determine the PSFs.

### Absorbed dose calculation

Convolution of the reconstructed ^99m^Tc SPECT data with the calculated function, $p_{Tc99m\to Y90}$ (equation 19), was applied to convert the resolution of the image to that of an ^90^Y SPECT scan. ^90^Y absorbed dose maps were calculated from the uncorrected ^99m^Tc, RCM ^99m^Tc, and ^90^Y SPECT data, under the assumptions of local energy deposition [24] and physical decay. The absorbed dose, D, in Gray (Gy) is given by:

(20) $$D=A_{0}\frac{T_{1/2}\sum n_{i}E_{i}\phi_{i}}{\ln\left(2\right)m}$$

For ^90^Y:

$n_{i}$ is the number of emissions per disintegrations, $n_{i}=1$ for the emission of beta particles

${\overset{-}{E}}_{i}$ is the average energy per beta emission (0.933 MeV) [25]

$\phi_{i}$ is the absorbed fraction. The local deposition method assumes the source is equal to the target, hence$\phi_{i}=1$

$T_{1/2}$ is the physical half-life (64.1 hours)

$A_{0}$ is the administered activity of ^90^Y (Bq)

m is the mass (kg)

Entering these constants into equation 20, and using $m_{\mathrm{voxel}}$ (the mass of a voxel in g) then gives the absorbed dose per voxel ($D_{\mathrm{voxel}}$) in Gy:

(21) $$D_{\mathrm{voxel}}=\frac{49.8\;A_{\mathrm{voxel}}}{m_{\mathrm{voxel}}}$$

where $A_{\mathrm{voxel}}$ is the activity per voxel in MBq. All absorbed dose maps were calculated using code written in IDL (Interactive Data Language, version 8.4, Exelis Visual Information Solutions, Inc.).

The counts within each voxel were converted to activity, $A_{\mathrm{voxel}}$, using a calibration factor ($C_{\mathrm{voxel}}$) calculated from the total counts ($C_{\mathrm{total}}$) in the phantom and the ^90^Y administered activity ($A_{0}$) [26]:

(22) $$A_{\mathrm{voxel}}=C_{\mathrm{voxel}}\left(\frac{A_{0}}{C_{\mathrm{total}}}\right)$$

The total counts in the phantom were obtained from a volume of interest (VOI) manually outlined on the CT scan marked by the phantom boundaries.

### Analysis

The spheres within the phantom were outlined manually using the CT image. VOIs delineated on the CT image were transcribed to the registered absorbed dose maps. The voxels of the dose map representing the background compartment of the phantom were identified by subtracting the sphere VOIs from the phantom VOI. The mean absorbed doses to the background compartment and spheres were then calculated.

Comparison of the absorbed doses to the spheres and background volumes, derived from ^99m^Tc and ^90^Y SPECT, was performed by calculation of the percentage differences (%ΔD) between them according to:

(23) $$\%\Delta D=\frac{D_{{99m}_{\mathrm{Tc}}}-D_{90_{Y}}}{D_{90_{Y}}}\times 100$$

where $D_{{99m}_{\mathrm{Tc}}}$ is the mean ^90^Y absorbed dose derived from the ^99m^Tc SPECT and $D_{Y90}$ is the mean ^90^Y absorbed dose derived from the ^90^Y SPECT. The percentage difference in absorbed doses derived from the ^99m^Tc SPECT after the application of the RCM and the ^90^Y SPECT were also calculated:

(24) $$\%\Delta D=\frac{D_{{99m}_{\mathrm{Tc}⊗p_{Tc99m\to Y90}}}-D_{90_{Y}}}{D_{90_{Y}}}\times 100$$

where $D_{{99m}_{\mathrm{Tc}⊗p_{Tc99m\to Y90}}}$ is the mean ^90^Y absorbed dose derived from the RCM ^99m^Tc SPECT image.

Uncertainties in the absorbed dose comparisons were calculated using the law of propagation of uncertainty described within the Bureau International des Poids et Mesures (BIPM) Guide to expression of uncertainty in measurement [27] and detailed by Gear et al [28].

## Clinical study

### Patient characteristics

The RCM was applied to SPECT-CT data in a retrospective study of 16 patients who had undergone ^90^Y resin microsphere treatment. All patients had a contrast enhanced CT (CECT) scan between 12 and 141 days prior to treatment, and no other liver treatments were performed between the CECT and SIRT. The patients had a range of primary tumour sites (colorectal, oesophago-gastric junction, breast, pancreatic and cholangiocarcinoma) with tumour burden ranging from 1% to 44%. Administered ^90^Y activity ranged from 900 to 2320 MBq.

### Planning and administration of SIRT

All patients were treated following pre-therapy angiography to evaluate the hepatic vasculature and to identify and embolise any aberrant vessels that may have led to extra-hepatic uptake of the microspheres. ^99m^Tc-MAA was administered and planar imaging performed to assess the lung shunt, followed by SPECT-CT imaging. The ^90^Y resin microsphere activity was calculated using the body surface area (BSA) method:

(24) $$A_{0}=\left(BSA-0.2\right)+\left(\frac{V_{\mathrm{tumour}}}{V_{\mathrm{tumour}}+V_{\mathrm{normal}\;\mathrm{liver}}}\right)$$

where $V_{\mathrm{tumour}}$ is the volume of the tumour and $V_{\mathrm{normal}\;\mathrm{liver}}$ is the volume of the normal liver. Both parameters were determined by manual delineation of the liver and tumours on the diagnostic CECT scan. The BSA was calculated using the DuBois and DuBois method [29].

The administration of the ^90^Y microspheres was performed by slow infusion, and ^90^Y bremsstrahlung SPECT-CT imaging was performed directly after treatment (Table 1). Acquisition data were reconstructed using the same reconstruction parameters as for the phantom study.

### Absorbed dose calculation

The RCM was applied to the ^99m^Tc SPECT patient images. Reconstructed SPECT data were registered to the CECT scans using automatic rigid registration in Hermes Hybrid Viewer, followed by manual adjustment if necessary. Liver and tumour VOIs were manually delineated on the CECT. Tumours below 2.6 cm^3^ and those with no uptake on the pre-therapy scan were excluded. ^90^Y absorbed dose maps were calculated from the uncorrected ^99m^Tc, RCM ^99m^Tc, and ^90^Y SPECT images, as described in equation 21, using a patient-specific calibration factor (equation 22) where the total counts were obtained from the liver VOI.

### Analysis

The tumour and liver VOIs were transcribed to the absorbed dose maps and manually adjusted if any clear mis-registration was visible. Mean absorbed doses to the tumours and normal liver were calculated, and voxel dose values within the normal healthy liver were used to calculate dose volume histogram (DVH) metrics: D50 (minimum absorbed dose value received by 50% of the normal liver) and D70 (minimum absorbed dose value received by 70% of the normal liver). The percentage differences in mean absorbed doses, D50 and D70, derived from predicted and delivered absorbed dose maps were calculated as previously defined (equations 23 and 24). Bland-Altman analysis was also performed. Percentage differences were also plotted as a function of tumour size. Uncertainties in the absorbed dose comparisons were calculated as for the phantom calculations.

## Results

### Measurement of point spread functions

**Fig. 1:**
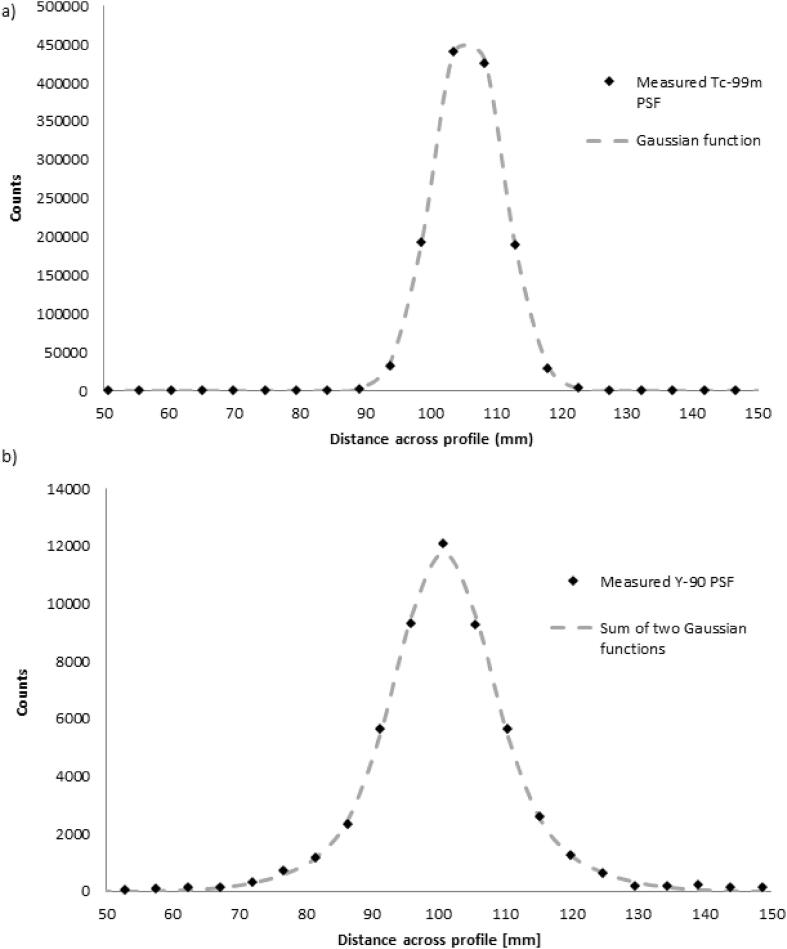
A) example of ^99m^Tc PSF with a Gaussian function fitted to it and B) example of ^90^Y PSF with the sum of two Gaussian functions fitted to it.

The x, y and z FWHM values for ^99m^Tc were 9.8, 12.7 and 12.2 mm respectively. An average value of 11.6 mm was calculated with a standard deviation of$\sigma_{Tc99m}$ = 4.9 mm. An example of a ^99m^Tc PSF with a Gaussian function fitted to it can be seen in Fig. 1a.

The sum of two Gaussian functions was fitted to the 3-dimensional ^90^Y PSF ($p_{Y90})$ which resulted in FWHM values of 19.2 mm ($p_{{Y90}_{1}}$) and 86.4 mm ($p_{{Y90}_{2}}$). This gave standard deviations of $\sigma_{{Y90}_{1}}$ = 8.2 mm and $\sigma_{{Y90}_{2}}$ = 36.7 mm, with $A_{1}$ = 0.34 and $A_{2}$ = 0.66. An example of a ^90^Y PSF with the sum of two Gaussian functions fitted to it can be seen in Fig. 1b.

The standard deviations of the function ($p_{Tc99m\to Y90}$) used in the RCM were then calculated from equations 17 and 18 as $\sigma_{{Tc99m\to Y90}_{1}}$ = 6.5 mm and $\sigma_{{Tc99m\to Y90}_{2}}$ = 36.4 mm.

## Phantom study

### Phantom background

Comparison between the absorbed doses to the phantom background, calculated from the ^99m^Tc and ^90^Y SPECT scans, gave a range of differences from −0.5% to 3.6%. After the application of the RCM the range of differences was 0.1% to 3.3%.

### Phantom spheres

The mean absorbed dose to each of the three smallest spheres (all less than 2.6 cm^3^) was less than one standard deviation above the mean absorbed dose delivered to the background compartment for all four of the ^90^Y phantom SPECT scans, rendering them undetectable. Therefore these were excluded from the analysis.

**Fig. 2:**
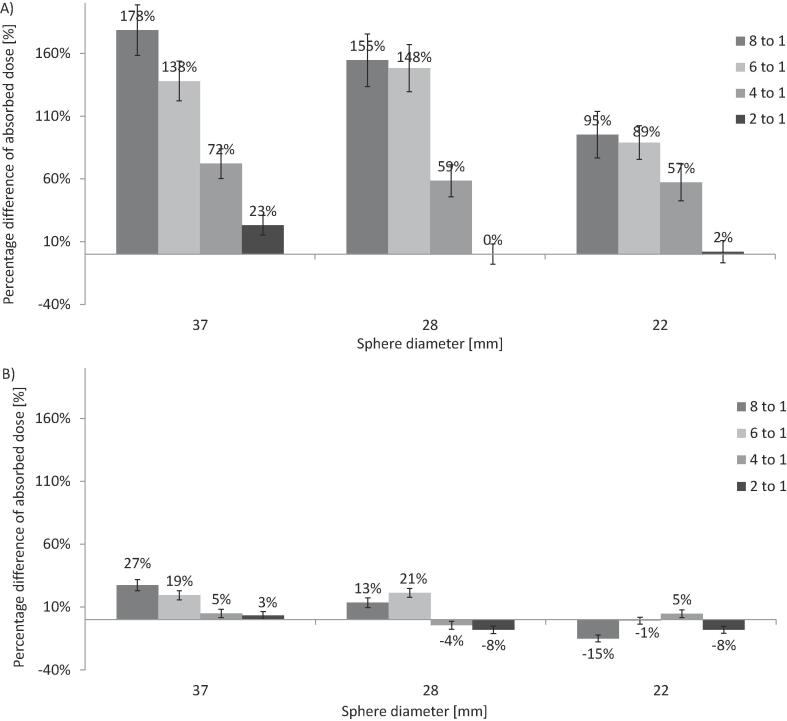
Percentage difference of predicted and delivered ^90^Y absorbed doses to the phantom spheres A) before and B) after application of the RCM. The different shades represent the different sphere-to-background ratios.

A wide range of differences (0 to 178%) between sphere absorbed doses derived from ^99m^Tc and ^90^Y SPECT data was obtained, (Fig. 2). After the application of the RCM the differences ranged from −15% to 27%.

## Clinical study

### Normal liver

**Fig. 3:**
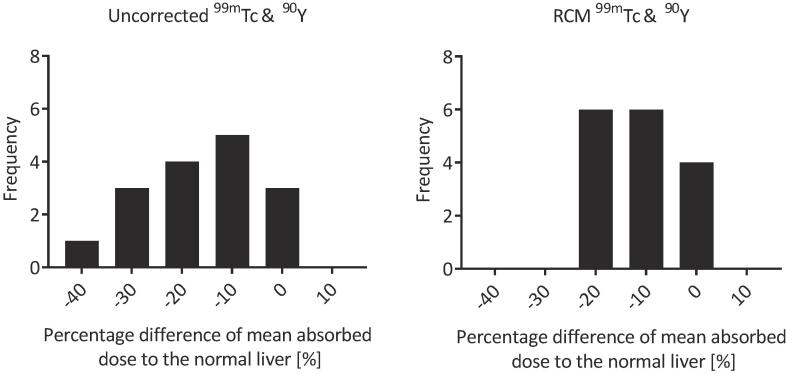
Histograms of the percentage difference between predicted and delivered mean absorbed dose to the normal liver A) before and B) after application of the RCM. Bin size is 10%.

**Fig. 4:**
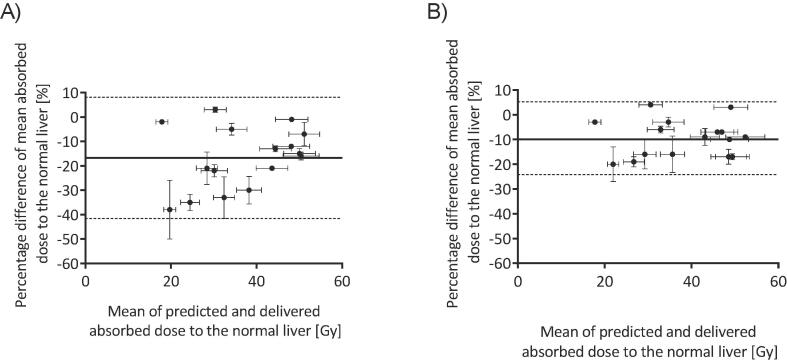
Bland-Altman analysis of predicted versus delivered absorbed doses to normal liver A) before and B) after application of the RCM. The bias shown by the solid line was −17%, reduced to −10% after the application of the RCM. The 95% confidence intervals are represented by the dashed lines.

The range of percentage differences between predicted and delivered absorbed doses were −38% (9 Gy) to 3% (1 Gy) (Fig. 3). Bland-Altman analysis is given in Fig. 4 where a bias of −17% was calculated. After the application of the RCM the percentage differences between the predicted and delivered mean absorbed doses were reduced to a range of −20% (5 Gy) to 4% (1 Gy) (Fig. 3) and a bias of −10% was found.

The percentage differences between the predicted and delivered D50 values ranged from −75% to −10%, and decreased to −31% to 4% after the application of the RCM. The percentage differences between the predicted and delivered D70 ranged from −91% to –33%, and decreased to −43% to 11% after the application of the RCM. Graphs can be seen in the supplemental material.

### Tumours

**Fig. 5:**
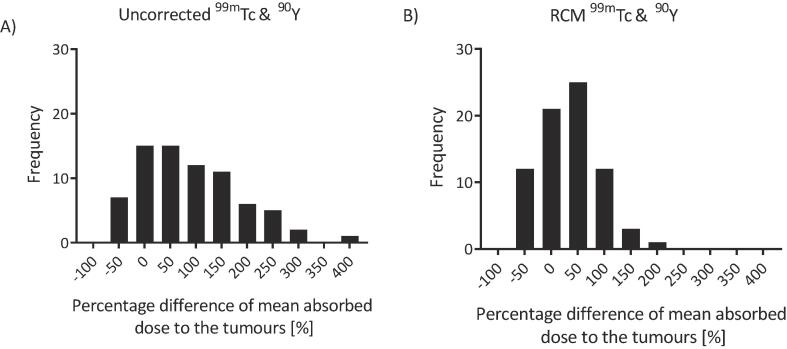
Histograms of the percentage difference between predicted and delivered absorbed dose to the tumours A) before and B) after application of the RCM.

Seventy-four tumours were analysed in total with a range in volume of 2.7 cm^3^ to 763.6 cm^3^ (equivalent diameters of 17.2 mm to 113.4 mm). The percentage differences between predicted and delivered absorbed doses to the tumours ranged from −49% (36 Gy) to 424% (205 Gy) (Fig. 5). The maximum absolute difference in absorbed dose was 216 Gy (195%).

After application of the RCM the percentage differences of the tumour absorbed doses ranged from −55% (41 Gy) to 210% (59 Gy) (Fig. 5). The maximum absolute difference between predicted and delivered absorbed doses to the tumours was 78 Gy (161%). No correlation was found between the percentage difference and the number of days between the CECT and the SIRT (r = 0.09, p = 0.5). Examples of pre- and post-therapy SPECT-CT scans with good and poor agreement are included in the supplemental material.

**Fig. 6:**
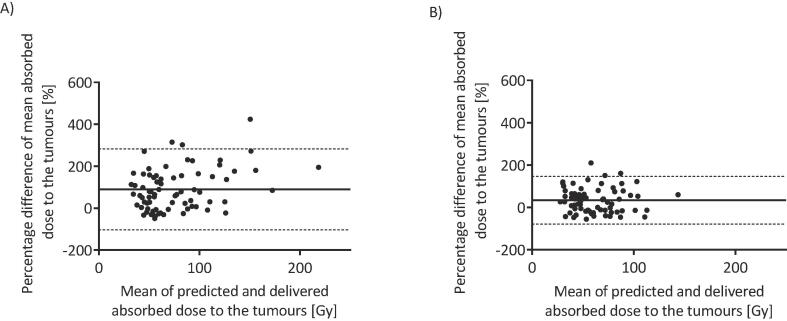
Bland-Altman analysis of predicted versus delivered absorbed doses to tumours A) before and B) after application of the RCM. The bias was shown by the solid line as 90%, reduced to 34% after the application of the RCM. The 95% confidence intervals are represented by the dashed lines.

Bland-Altman analysis (Fig. 6) demonstrated no relationship between the mean absorbed doses to the tumours and the percentage differences. A bias of 90% was found, which indicates that the predicted absorbed doses to the tumours were higher than the delivered absorbed doses. This was reduced to 34% after the RCM was applied.

**Fig. 7:**
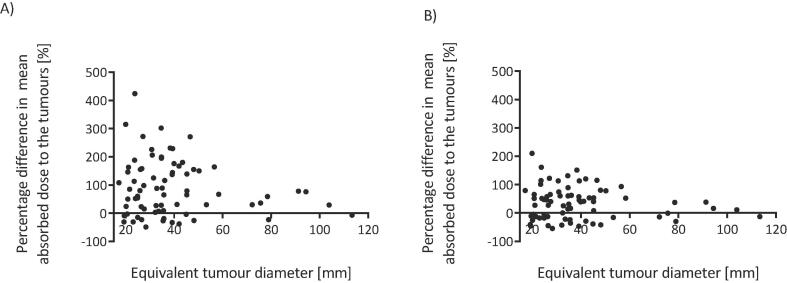
Percentage difference between predicted and delivered absorbed doses to the tumours plotted against tumour equivalent diameter A) before and B) after application of the RCM. nce was 271%, and 115% after the RCM was applied.

A larger range of percentage differences in tumour absorbed doses was demonstrated for smaller tumours than for larger tumours (Fig. 7). The range of percentage differences for tumours less than 100 cm^3^ (equivalent diameter 57.6 mm) was −49% to 424% before, and −55% to 210% after the RCM was applied. This was –23% to 67% before and −30% to 52% after the application of the RCM, for tumours greater than 100 cm^3^.

## Discussion

SIRT has been shown to be a successful treatment demonstrating good tumour response [30], [31], [32] and increasing time to further hepatic progression [32], [33], [34], [35]. Prospective treatment planning with pre-therapy ^99m^Tc-MAA SPECT has demonstrated a good correlation between tumour dose and response [35], [36], [37]. However, studies have shown contrasting findings when investigating the predictive power of the pre-therapy ^99m^Tc-MAA distribution [16], [17], [19], [20], [38], [39], [40]. To allow direct quantitative comparison between predicted and delivered ^90^Y absorbed dose, a novel RCM method was developed to address the differences in partial volume effects between ^99m^Tc-MAA SPECT and ^90^Y bremsstrahlung SPECT images by converting the spatial resolution of the pre-therapy scan data to that of the post-therapy scan data. The method was validated on phantom scans and then applied retrospectively to a set of patient data. A similar method has also been proposed by Mikell et al [21] who blurred the ^90^Y PET to match the ^99m^Tc SPECT.

Point spread functions for ^99m^Tc and ^90^Y SPECT were measured experimentally and used as inputs into the RCM. The ^90^Y PSF was fitted to a mathematical model of the sum of two Gaussian functions to incorporate the large septal penetration tails. The PSF’s for ^131^I [41] and ^123^I [42] have previously been fitted with a Gaussian and an exponential function to account for septal penetration tails in the collimator detector response compensation.

The phantom study validated the use of the RCM and established differences between predicted and delivered ^90^Y absorbed doses due to measurement uncertainties. Deviations observed in the clinical analysis were considered due to either biological differences in the particle distributions between the pre-and post-clinical scans caused by differences in particle size or catheter positioning, or due to limitations of the phantom study. Regions within the phantom only allowed for uniform activity distributions. In addition, the individual sphere volumes, and the total sphere volume, were small compared to many of the tumours identified in the clinical study. Comparison of the predicted and delivered ^90^Y absorbed doses demonstrated small differences before and after the application of the RCM for the phantom background. Analysis of the mean absorbed doses in the phantom spheres identified large differences of up to 178%; these were reduced to 27% with the application of the RCM.

Analysis of the patient data demonstrated the predictive power of the ^99m^Tc-MAA pre-therapy imaging, with differences between predicted and delivered mean absorbed dose to the normal liver reduced from 38% to less than 20% after the application of the RCM. This is larger than the differences demonstrated in the validation for the phantom background and could be due to either the limitations of the phantom, biological differences in the particle distributions or due to tumoural uptake from the SPECT scan included within the normal liver VOIs. The DVH metrics demonstrated less agreement than the mean absorbed dose; they were reduced from 75% to 31% for D50, and from 91% to 43% for D70.

This study suggests that pre-therapy imaging could be used for prospective treatment planning to provide an estimate of the delivered mean absorbed dose to the normal liver within 20%. A previous comparison demonstrated good concordance between predicted and delivered absorbed dose to the normal liver [20]. This is in contrast with the study by Wondergem et al [14] where analysis demonstrated larger absorbed dose differences in segments with smaller tumour involvement leading to the assumption that the differences were due to the normal liver uptake. A study by Kafrouni et al [39] also found significant differences between predicted and delivered ^90^Y normal liver absorbed doses, although absolute and percentage differences were not reported.

The application of the RCM resulted in a reduction of both the percentage and absolute differences between the predicted and delivered absorbed doses to the tumours, as well as a reduction in the bias from 90% to 34%. However, a wide range of differences were still present after the RCM was applied. In this study 41% of tumours (after application of the RCM) had an absorbed dose difference above 50%. This is in agreement with Willowson et al [38] who found that 40% of tumours demonstrated a difference between predicted and delivered absorbed dose of more than 50%. No significant differences in tumour absorbed dose were found by Kafrouni et al [39], although values for specific tumours were not detailed. A large variation in absorbed dose differences was found for tumours less than 100 cm^3^ (equivalent diameter 57.6 mm). This is in agreement with Gnesin et al [20] who also demonstrated large variability for tumours less than 150 cm^3^ with an over-estimation in the predicted absorbed dose. It is not clear why there is such a large variation for small tumours even after accounting for differences in the partial volume effect with the application of the RCM. However, small tumours are often not clearly visible on the ^90^Y bremsstrahlung SPECT scans. This could result in greater misregistration errors and in turn lead to larger errors on the absorbed doses. The embolic effect of the resin microspheres may be more pronounced for smaller tumours with less vasculature surrounding them, resulting in different MAA and microsphere distributions. This hypothesis is supported by a previous study where glass microspheres were used preferentially over resin microspheres for small tumours to prevent tumour saturation and reflux into the normal liver [20]. It is also worth noting that the RCM method results in a degradation of the ^99m^Tc SPECT image, thus for small tumours with low intensity the signal may be lost.

There are some limitations to this study. It is well known that the PSF varies across the field of view [43]; in this work one PSF was used for the whole field of view. This could lead to uncertainties in the application of the RCM as demonstrated by comparison of the absorbed doses to the spheres where differences of up to 27% were observed. ^90^Y bremsstrahlung imaging is challenging, and in this work scatter and collimator detector response corrections were not performed during ^90^Y reconstruction which will limit the quantitative accuracy [2], [3]. It is possible that there was some degree of misregistration between the clinical SPECT and CECT scans. The time gap between these scans could result in changes to the tumoural liver anatomy between scans. Patient positioning for the CECT, ^99m^Tc SPECT and ^90^Y SPECT scans could also be different, leading to further misregistration which will result in the CECT VOIs being incorrectly placed on the SPECT scans and could lead to larger errors for smaller tumours. To minimise these errors, SPECT scans were manually adjusted after automatic registration. The position of VOIs was also manually adjusted if a clear mismatch was visible. Delineation was performed on the CECT scan. This method was chosen specifically to ensure analysis of identical tumour and normal liver areas between the pre-and post-therapy SPECT scans. However, it is accepted that this may have led to large differences in the comparison of the uncorrected SPECT scans due to different spatial resolutions. An alternative method would have been to use a thresholding method to define the VOIs. However, this method comes with its own disadvantages. The use of threshold VOIs is challenging with the optimal VOI choice dependant on tumour size as well as tumour to normal liver contrast. The advantage of the RCM is that it can be applied globally to a heterogeneous distribution of tumours. The PSFs fitted in this study are acquisition and reconstruction parameter dependent, therefore reproduction of this work would involve centres undertaking their own measurements.

## Conclusions

A resolution conversion method (RCM) was developed to overcome the differences in partial volume effects between predicted and delivered absorbed dose distributions. After initial validation with phantom data this was applied retrospectively to patient data. The analysis of this clinical data demonstrated that the RCM reduced the differences in the mean absorbed dose to the normal liver and led to the conclusion that the ^99m^Tc pre-therapy imaging was predictive of the ^90^Y mean absorbed dose to the normal liver to within 20%. However, although a reduction in the differences between the predicted and delivered mean absorbed doses to tumours was seen after the application of the RCM, the ^99m^Tc pre-therapy imaging had poor predictability for tumours smaller than 100 cm^3^.

## Ethics approval and consent to participate

All procedures performed in studies involving human participants were in accordance with the ethical standards of the institutional and/or national research committee and with the 1964 Helsinki Declaration and its later amendments or comparable ethical standards. This study utilised anonymised patient data collected as standard routine protocol only, and as such ethics committee approval was not required. All patients gave written informed consent.

## Consent for publication

Not applicable.

## Availability of data and material

Sharing of data with outside investigators is not permitted under the informed consent obtained from participants included in this study. The supporting data cannot be shared.

## Funding

Allison J Craig was funded by a National Institute for Health Research and Health Education England Healthcare Science Doctoral Research Fellowship.

## Authors contributions

AC, IM, AD and GF designed the study. BR, LH, AM and NK acquired the data. AC performed the data analysis and drafted the manuscript. IM, AD and GF supervised the project, helped with the analysis and edited the manuscript. BR assisted with the data analysis. JG and SC assisted with the data analysis and contributed to editing of the manuscript. All authors read and approved the final manuscript.

## Declaration of Competing Interest

The authors declare that they have no known competing financial interests or personal relationships that could have appeared to influence the work reported in this paper.
