# Supplementary material for: Comparison of 90Y SIRT predicted and delivered absorbed doses using a PSF conversion method
Source: Phys Med. 2021 Sep;89:1–10. doi: 10.1016/j.ejmp.2021.07.026 (PMC8501309; doi:10.1016/j.ejmp.2021.07.026)
Supplement: Supplementary data 1 [file mmc1.docx]

**Supplemental Figure 1: Histograms of the percentage difference between normal liver predicted and delivered D50 A) before and B) after application of the RCM, and D70 C) before and D) after application of the RCM. Bin size is 10%.**
