# Supplementary material for: Comparison of 90Y SIRT predicted and delivered absorbed doses using a PSF conversion method
Source: Phys Med. 2021 Sep;89:1–10. doi: 10.1016/j.ejmp.2021.07.026 (PMC8501309; doi:10.1016/j.ejmp.2021.07.026)
Supplement: Supplementary data 2 [file mmc2.docx]

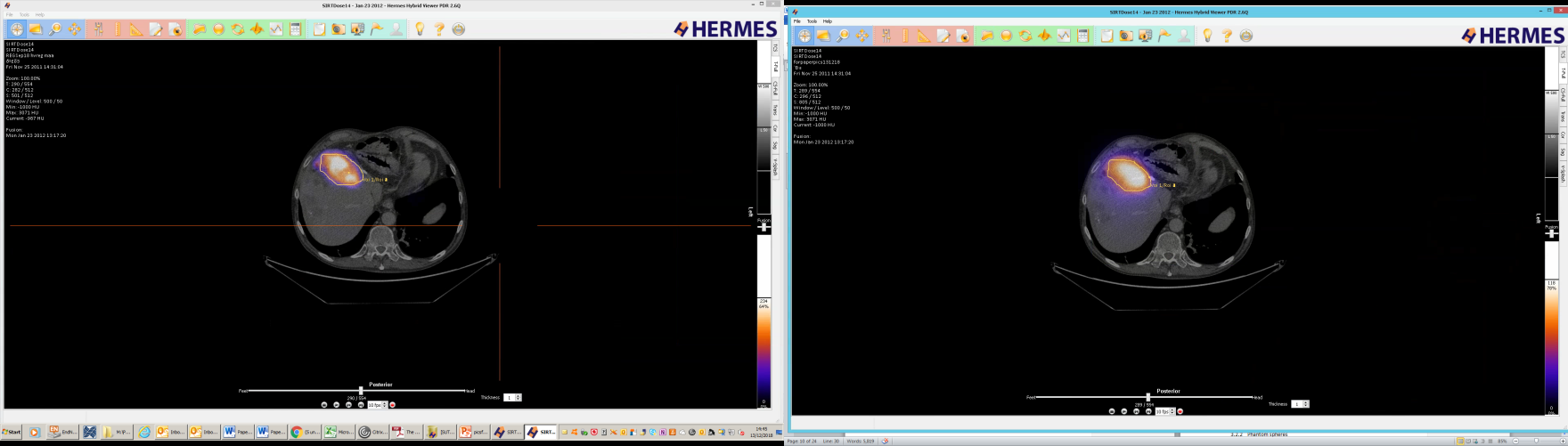

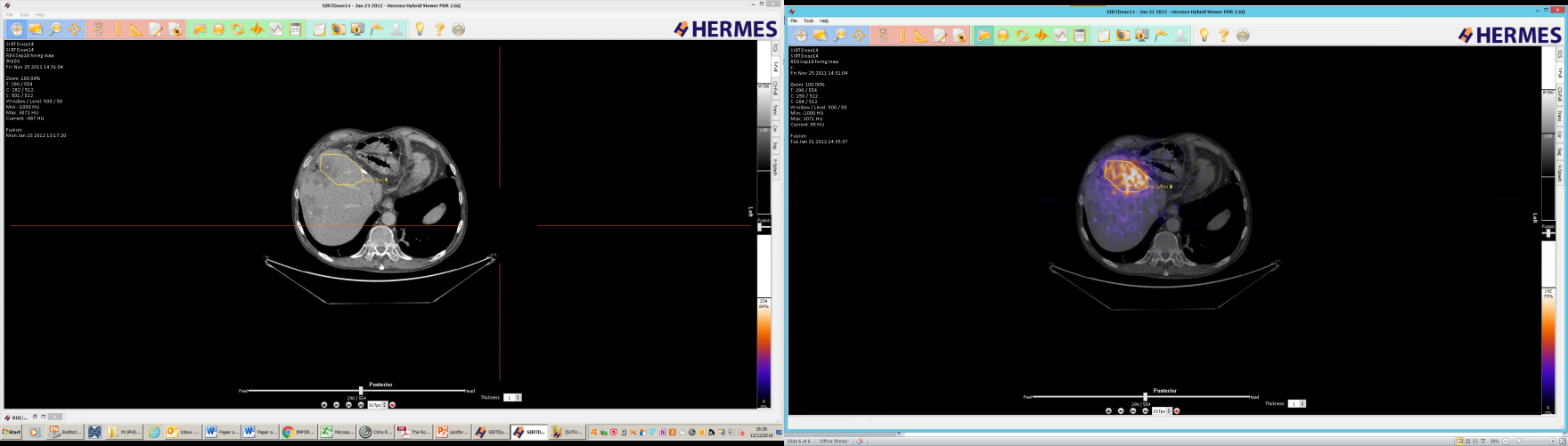

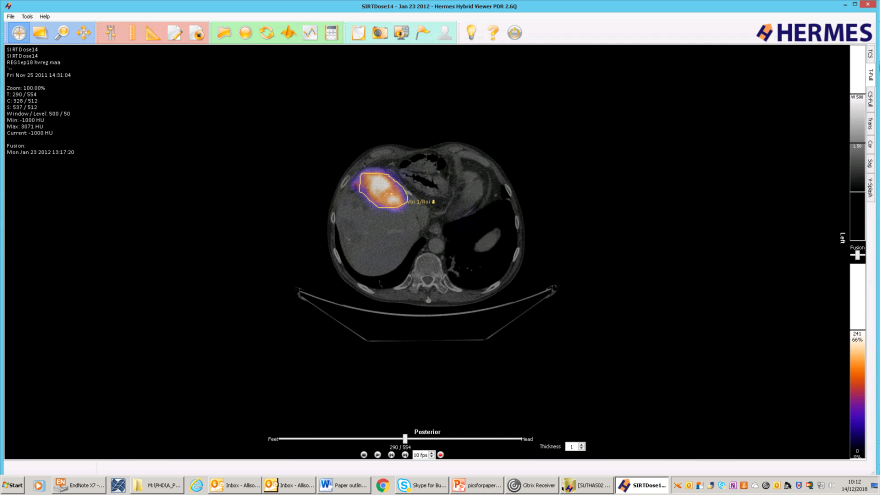


A)

B)

C)

Supplemental Figure 2: A) Pre-therapy SPECT-CT, B) pre-therapy SPECT-CT after application of the RCM and C) post-therapy SPECT-CT. The difference was 76%, and 16% after the RCM was applied.


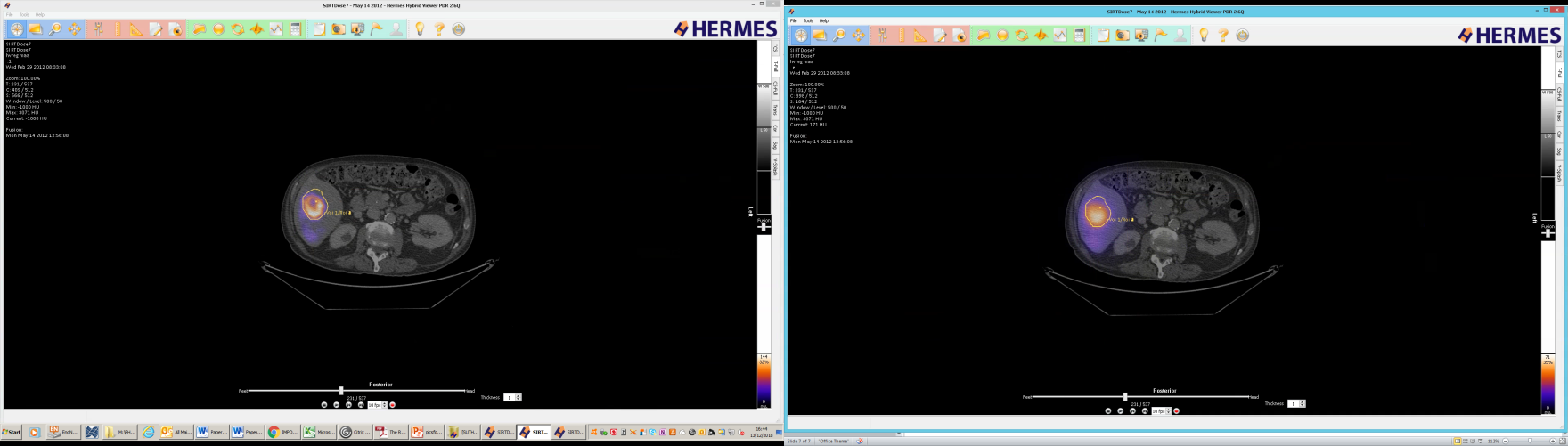

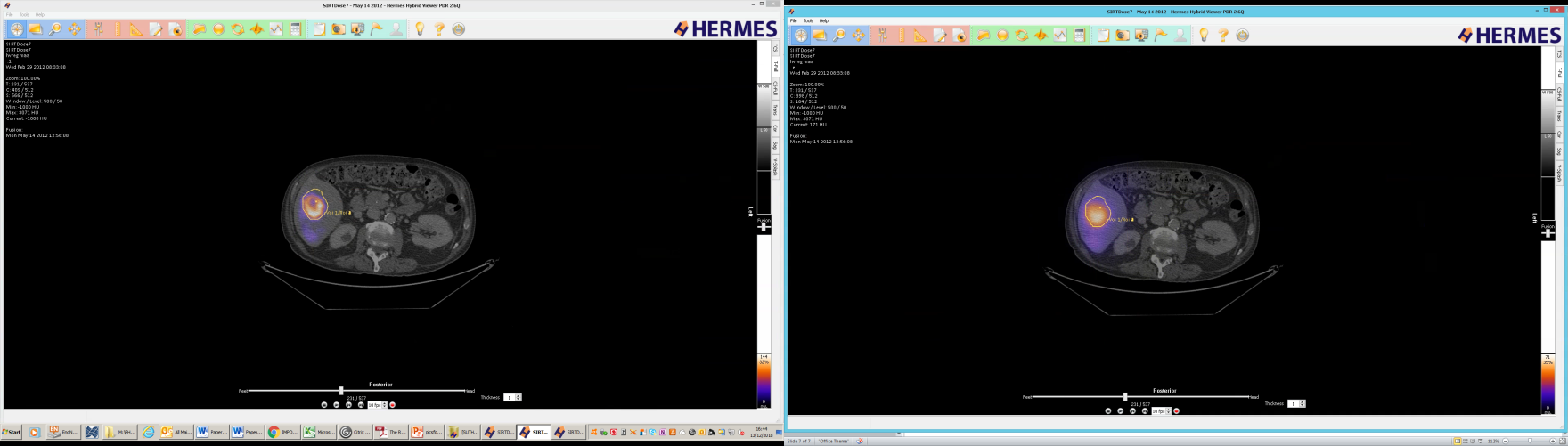

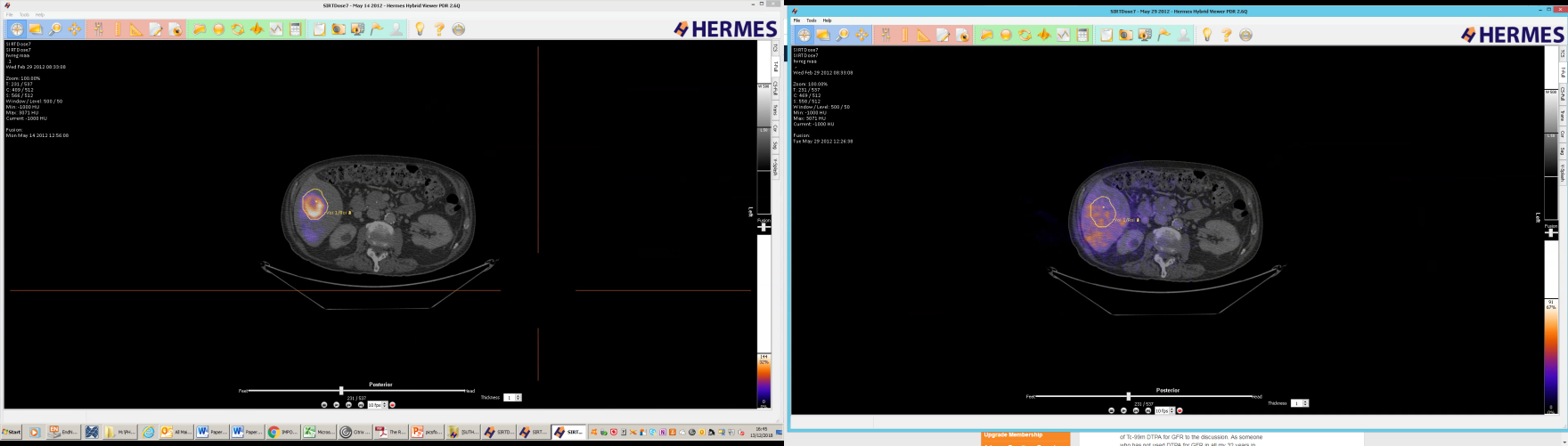


A)

B)

C)

Supplemental Figure 3: A) Pre-therapy SPECT-CT, B) pre-therapy SPECT-CT after application of the RCM and C) post-therapy SPECT-CT. The difference was 271%, and 115% after the RCM was applied.

C)
